# Emerging pharmacological tools to control hydrogen sulfide signaling in critical illness

**DOI:** 10.1186/s40635-020-0296-4

**Published:** 2020-01-31

**Authors:** Eizo Marutani, Fumito Ichinose

**Affiliations:** Department of Anesthesia, Critical Care and Pain Medicine, Massachusetts General Hospital and Harvard Medical School, Boston, MA 02114 USA

**Keywords:** Hydrogen sulfide, Sulfide synthesis, Sulfide catabolism, Sodium thiosulfate, Critical illness

## Abstract

Hydrogen sulfide (H_2_S) has long been known as a toxic environmental hazard. Discovery of physiological roles of H_2_S as a neurotransmitter by Kimura and colleagues triggered an intensive research in the biological roles of H_2_S in the past decades. Manipulation of H_2_S levels by inhibiting H_2_S synthesis or administration of H_2_S-releasing molecules revealed beneficial as well as harmful effects of H_2_S. As a result, it is now established that H_2_S levels are tightly controlled and too much or too little H_2_S levels cause harm. Nonetheless, translation of sulfide-based therapy to clinical practice has been stymied due to the very low therapeutic index of sulfide and the incomplete understanding of endogenous sulfide metabolism. One potential strategy to circumvent this problem is to use a safe and stable sulfide metabolite that may mediate effects of H_2_S. Alternatively, endogenous sulfide levels may be controlled using specific sulfide scavengers. In this review article, the role of endogenous H_2_S production and catabolism will be briefly reviewed followed by an introduction of thiosulfate and H_2_S scavengers as novel pharmacological tools to control H_2_S-dependent signaling.

## Background

Hydrogen sulfide (H_2_S) is a colorless gas with characteristic rotten egg odor, which has long been known as a toxic environmental pollutant [1]. Recently, H_2_S has emerged as an important gaseous signaling molecule that is generated endogenously in tissues along with nitric oxide (NO) and carbon monoxide (CO) [2–4]. In 1996, Abe and Kimura reported a physiological role of H_2_S as a neurotransmitter and identified cystathionine β-synthase (CBS) as an H_2_S-producing enzyme [5]. Intensive research thereafter revealed a number of physiological roles of H_2_S including vasodilation, angiogenesis, anti/pro-inflammation, oxygen (O_2_) sensing, and cytoprotection [6–8]. These studies showed that endogenous H_2_S metabolisms (production and catabolism) play critical roles both in normal physiology and in some human disorders. Manipulation of H_2_S levels by inhibiting H_2_S synthesis or administration of H_2_S-releasing molecules revealed beneficial as well as harmful effects of H_2_S [9–14]. Too much or too little H_2_S levels appear to cause harm. For example, deficiency in CBS or another H_2_S-synthesis enzyme cystathionine γ-lyase (CSE or CTH) causes hypertension in mice [15, 16]. While high-dose sodium sulfide (Na_2_S), a H_2_S-donating compound, exaggerates ischemic brain injury, low-dose Na_2_S or inhibitors of CSE or CBS decreases ischemic stroke size [17]. Deficiency in CSE promotes neurodegeneration in Huntington’s disease [18], whereas deficiency in ethylmalonic encephalopathy 1 (ETHE1 or persulfide dioxygenase, PDO), a H_2_S catabolizing enzyme, is a cause of ethylmalonic encephalopathy, which is characterized by abnormally high H_2_S levels in tissues and blood [19]. These observations indicate that dysregulated H_2_S metabolism may be pathogenic. However, controlling sulfide levels has proven to be very difficult using chemical H_2_S donors or inhibitors of H_2_S-producing enzymes. In recent studies, we revealed thiosulfate, an oxidative metabolite of H_2_S, may hold promise as a low toxicity sulfide donor. We also developed specific H_2_S scavenger to control local concentration of H_2_S. We will review the role of endogenous H_2_S production and catabolism followed by a focused discussion of thiosulfate and H_2_S scavengers as emerging pharmacological strategies to control H_2_S-dependent signaling.

## Endogenous H_2_S production

**Fig. 1:**
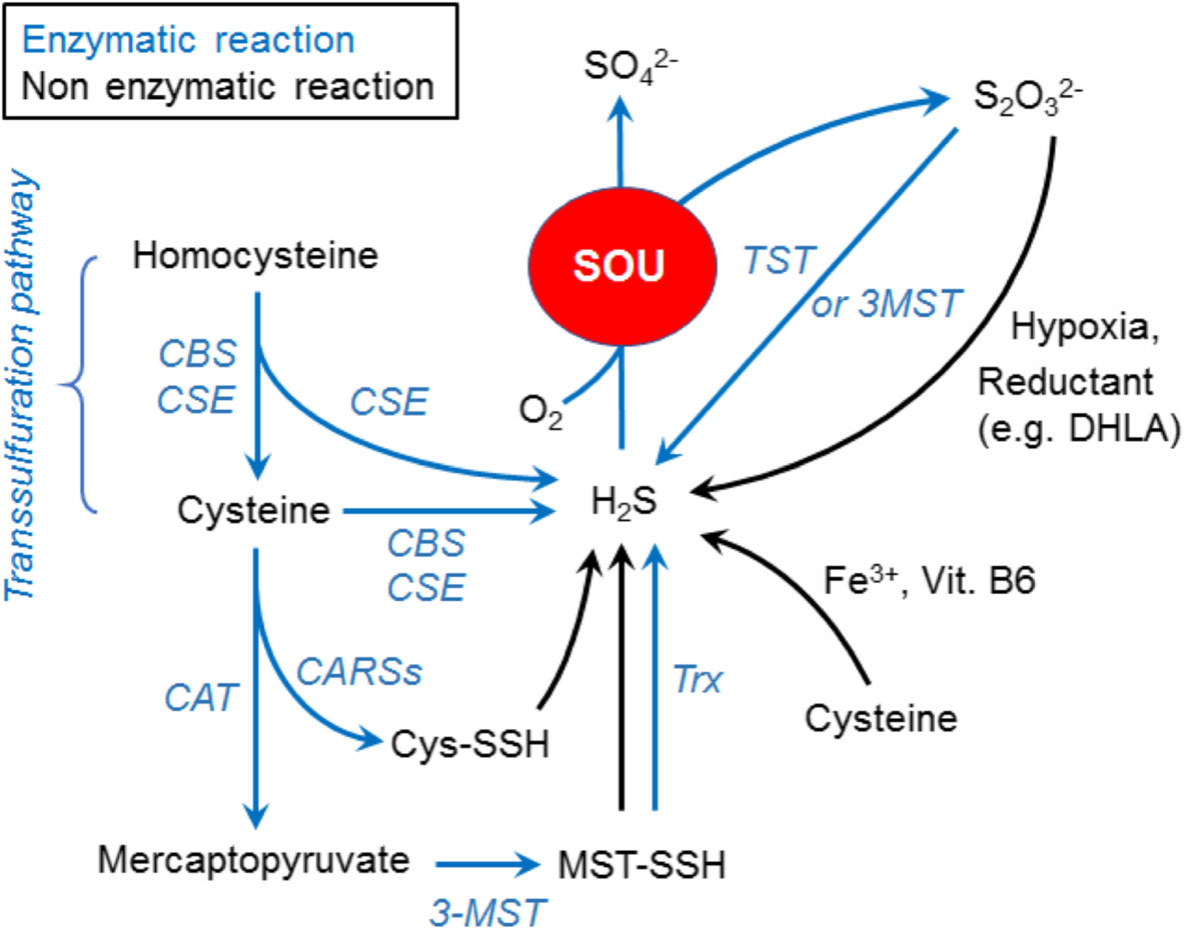
Pathways of H_2_S production. Cysteine is produced from homocysteine via transsulfuration pathway mediated by cystathionine β-synthase (CBS) and cystathionine gamma-lyase (CSE, CGL or CTH). H_2_S is produced from homocysteine and cysteine by CBS and CSE. 3-Mercaptopyruvate sulfurtransferase (3-MST) generates 3-MST-cysteine persulfide (MST-SSH) utilizing mercaptopyruvate which is produced from cysteine by cysteine aminotransferase (CAT). H_2_S is released from MST-SSH via non-enzymatic reaction or catalytic activity of thioredoxin (Trx). H_2_S is oxidized by sulfide oxidation unit (SOU) to produce thiosulfate and sulfate utilizing O_2_ as described in the “H_2_S catabolism” section. H_2_S is generated from thiosulfate by non-enzymatic reaction using reductants in hypoxia or catalytic activity of thiosulfate sulfurtransferase (TST) or 3-MST. H_2_S is non-enzymatically produced from cysteine in an iron (Fe^3+^)- and vitamin B_6_-dependent manner

Studies have revealed enzymatic and non-enzymatic H_2_S-producing pathways (Fig. 1). In enzymatic pathways, CBS, CSE, 3-mercaptopyruvate sulfurtransferase (3-MST), and cysteinyl-tRNA synthetase (CARS) contribute to endogenous production of H_2_S directly or indirectly [20–23]. Approximately one fifth of sulfide exists as hydrosulfide ion (HS^−^) and the remaining four fifth consist mostly of H_2_S with little amount of S^2−^ in physiological fluids (37 °C, pH 7.4) according to the Henderson-Hasselbalch equation [24].

The transsulfuration pathway is a metabolic pathway where transfer of sulfur from homocysteine to cysteine occurs [25]. Products of this pathway include various sulfur metabolites such as cysteine, glutathione, and H_2_S. CBS and CSE produce H_2_S from cysteine and homocysteine requiring a cofactor pyridoxal 5′-phosphate (PLP) via the transsulfuration pathway. CBS and CSE are mainly localized in cytosol while some reports suggest that CBS or CSE could translocate into mitochondria under hypoxic stress or conditions that increased intracellular free calcium, respectively [26, 27]. Driving catabolic H_2_S oxidation is significant electron source for electron transport chain (ETC) as described below in this review [28–30]. Translocation of CSE from cytosol to mitochondria is important to maintain ATP level in hypoxia in vascular smooth muscle cells [27]. These observations indicate the important role of H_2_S produced by CBS and CSE in maintenance of ATP production in hypoxia. Deficiency in CBS or CSE causes marked hyperhomocysteinemia and hypertension in mice [15, 16]. Disruption of CBS in mice causes metabolic osteoporosis that is prevented by supplementation of H_2_S [31]. Deficiency in CSE promotes neurodegeneration in Huntington’s disease [18]. CSE deficiency ameliorates acute liver failure (ALF) in mice [32].

3-MST is a sulfurtransferase that produces sulfane sulfur, a sulfur atom with six valence electrons, rather than H_2_S. 3-Mercaptopyruvate, a substrate of 3-MST, is produced by cysteine aminotransferase (CAT). 3-MST produces sulfane sulfur transferring sulfur in sulfane group of 3-mercaptopyruvate to other sulfur acceptor using zinc as a cofactor. H_2_S is subsequently released from sulfane sulfur [23]. 3-MST localizes both in cytosol and mitochondria. Thiosulfate sulfurtransferase (rhodanese or TST) is also known as a sulfurtransferase in mitochondria which produces sulfane sulfur although biological activity of this enzyme remains poorly understood [23]. Deficiency in 3-MST augments anxiety-like behavior in mice [33].

CARSs have been found initially as an enzyme that mediates translation of proteins in prokaryotes and eukaryotes including mammals [21]. CARS-1 and CARS-2 are localized in cytosol and mitochondria, respectively. Polysulfidation (or persulfidation) at the cysteine residue of proteins has been recognized as a “post”-translational protein modification which could modulate catalytic activity of the protein by altering protein conformations and/or directly changing the activity of catalytic centers [34–37]. Akaike et al. found that CARSs mediate polysulfidation of proteins “during,” but not post-, protein translation in a PLP-dependent manner. Because sulfane sulfur in protein polysulfide can be a source of H_2_S, CARS increases H_2_S levels indirectly. Homozygous disruption of CARS-2 is embryonic lethal [21]. Heterozygous disruption of CARS-2 decreases the tissue levels of persulfide, polysulfide, sulfide, and thiosulfate, while other phenotypes of CARS-2^+/−^ mouse remain to be elucidated because CARS knockout mice have been generated very recently [21].

Studies using inhibitors of H_2_S synthesizing enzymes have suggested the biological role of endogenous H_2_S production. However, most of the currently available inhibitors are far from ideal [38]. For example, dl-propargyl glycine (PAG or PGG) or aminooxyacetic acid (AOAA) is the most frequently used compound as a CSE or CBS inhibitor, respectively. Despite only l-isomer of PAG, but not d-isomer, inhibits CSE and d-isomer may be a nephrotoxin, many studies have used the mixture of both isomers [39–41]. PAG has been typically used in the range of 1–10 mM which is significantly higher than IC_50_ (40 μM) possibly because of the limited cell permeability [40]. At millimolar concentrations, PAG also inhibits other enzymes such as aspartate aminotransferase and alanine aminotransferase [42, 43]. Because IC_50_ of AOAA for CSE and CBS are in the similar range (2–8.5 μM and 1.1 μM, respectively), AOAA is not a specific CBS inhibitor [38]. More specific and less toxic inhibitors are required to examine precise roles of H_2_S synthesizing enzymes.

There have been a number of non-enzymatic H_2_S productions reported. Iron (Fe^3+^) generates H_2_S from cysteine in a vitamin B_6_- and pyridoxal (or PLP)-dependent manner [44]. Regulation of H_2_S production via this pathway may contribute to pathophysiology of conditions with iron dysregulation such as hemolysis, iron overload, and hemorrhagic disorders. H_2_S can also be generated from thiosulfate, one of H_2_S oxidation products, in the presence of an endogenous reductant (e.g., dihydrolipoic acid) without enzymes [45]. Interestingly, H_2_S production via thiosulfate is augmented in hypoxic conditions [45].

## H_2_S catabolism

**Fig. 2:**
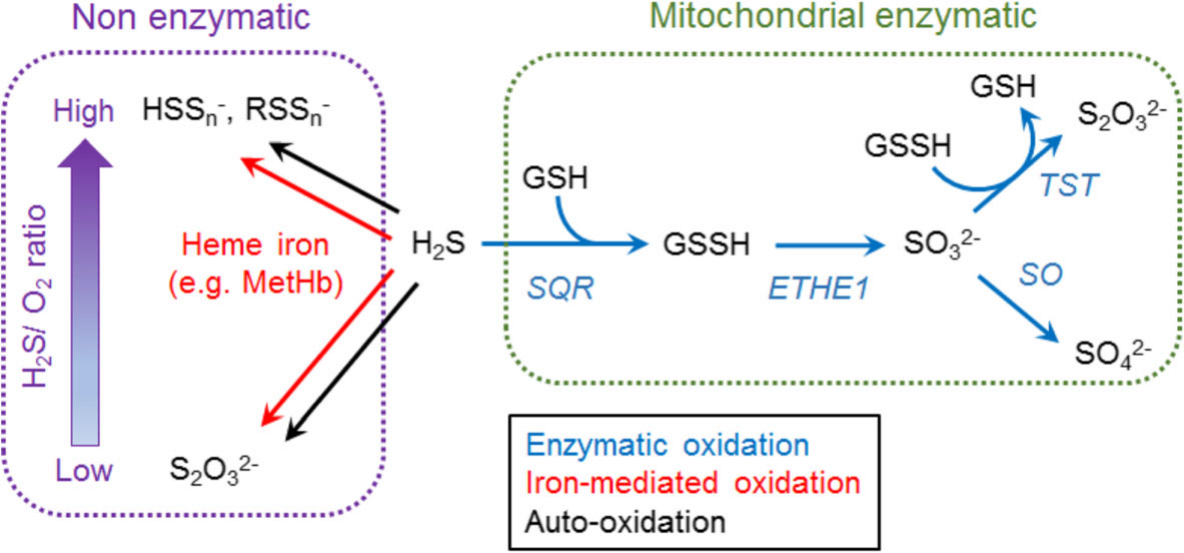
Pathways of H_2_S oxidation. H_2_S is serially oxidized to generate GSSH by sulfide: quinone oxidoreductase (SQR). Sulfur dioxygenase (ETHE1 or SDO) catabolizes GSSH to produce sulfite which is further catabolized to produce thiosulfate and sulfate by catalytic activity of thiosulfate sulfurtransferase (TST or rhodanese) and sulfite oxidase (SO), respectively. H_2_S can be oxidized by the heme iron-mediated oxidation or auto-oxidation to produce thiosulfate or polysulfide. The main oxidative product is thiosulfate or polysulfide when sulfide/O_2_ level is low or high, respectively

H_2_S is catabolized via both enzymatic and non-enzymatic pathways (Fig. 2). In enzymatic catabolism, H_2_S is oxidized serially by sulfide oxidation unit (SOU), a cluster of mitochondrial enzymes. SOU consists of sulfide: quinone oxidoreductase (SQR or SQOR), ETHE1 or sulfide dioxygenase (SDO), TST, and sulfite oxidase (SO) [30, 46]. H_2_S is oxidized by SQR to generate sulfane sulfur (S^0^) which has six valence electrons and no charge forming persulfide (SQRS-S). SQR utilizes the oxidized form of coenzyme Q (CoQ) as an electron acceptor through the H_2_S oxidation to produce the reduced form of CoQ which is consumed to drive ETC. As the next step, SQR transfers sulfane sulfur to sulfur acceptors such as glutathione (GSH), sulfite (SO_3_^2−^), cysteine, and homocysteine while SQR utilizes GSH as a dominant sulfur acceptor in the physiological conditions to produce glutathione persulfide (GSSH) which is the main product of H_2_S oxidation mediated by SQR. ETHE1 converts GSSH into sulfite and GSH consuming oxygen. Sulfite is further catabolized by TST or SO to produce thiosulfate (S_2_O_3_^2−^) or sulfate (SO_4_^2−^), respectively. Thiosulfate can be converted back to H_2_S via catabolism by 3-MST and TST [45].

Non-enzymatic H_2_S catabolic pathways play some roles. H_2_S undergoes auto-oxidation both in aerobic and anaerobic conditions. High or low sulfide/oxygen ratio results in polysulfide or thiosulfate production in the buffer at physiological pH, respectively [47]. H_2_S also binds to heme iron of methemoglobin (MetHb) to be converted into thiosulfate and polysulfide [48]. MetHb is formed by auto-oxidation of ferrous Hb and represents 1–3% of total Hb. Therefore, MetHb concentration is 25–75 μM in blood, which is significantly higher than circulating H_2_S level (~ 0.2 μM) [48, 49]. This observation indicates that red blood cells play a critical role in maintaining circulating H_2_S at physiologically low levels. Myoglobin can also exert similar capacity of H_2_S oxidation as MetHb to generate thiosulfate and polysulfide [50]. H_2_S can bind to other globin species (e.g., neuroglobin), which indicates the possible H_2_S oxidation by these globin species [51–53].

Impairment of sulfide catabolism could be pathogenic. For example, mutation in ETHE1 is responsible for ethylmalonic encephalopathy [19, 54–56]. Ethylmalonic encephalopathy is an autosomal recessive disorder that affects several body systems, particularly the nervous system. Neurological signs and symptoms include delayed development and developmental regression, muscle weakness (hypotonia), seizures, and abnormal movements. ETHE1-deficient mice exhibit cardinal features of ethylmalonic encephalopathy and die between the fifth and sixth weeks after birth. ETHE1-deficient mice show sulfide accumulation and deterioration of complex IV activity in tissues including the brain.

Deficiency in TST markedly exacerbates, whereas TST activation by thiosulfate administration ameliorates, diabetes in mice [57]. TST expression level in human adipose tissue is correlated positively with adipose insulin sensitivity and negatively with fat mass, suggesting TST activation may be beneficial for type II diabetes.

## Administration of H_2_S donor as therapeutic measure

The effects of administration of exogenous H_2_S were initially examined using simple sulfide salts (e.g., Na_2_S, NaHS). For example, intra-left ventricular administration of Na_2_S at 50 μg/kg attenuated myocardial ischemic injury by preserving mitochondrial function in mice [58]. Systemic administration of Na_2_S at 7 μmol/kg IV improves survival rate and attenuates brain injury after cardiac arrest and cardiopulmonary resuscitation in mice via nitric oxide synthase 3-dependent manner [59]. Systemically administered sulfide salts increase circulating H_2_S concentration instantly while H_2_S levels return to the baseline quickly due to the short half-life of H_2_S (shorter than 2 min in PBS and cell culture medium, around 4 min in the blood) [48, 60, 61]. Subsequently, a number of compounds that slowly release H_2_S after administration were developed [8, 38, 62]. GYY4137, a water-soluble slowly H_2_S-releasing compound, exerts beneficial effects of H_2_S even with wide therapeutic window (0.1–5 mM) and has been used frequently in both in vitro and in vivo experiments [63]. Because H_2_S-induced neurotoxicity may be mediated via enhancement of *N*-methyl-d-aspartate receptor (NMDAR) activation [64–66], we developed a novel hybrid H_2_S-releasing molecule, S-memantine, which is a combination drug of slowly H_2_S-releasing molecule chemically conjugated with memantine which is a moderate NMDAR antagonist and approved for the treatment of Alzheimer’s disease patients. S-memantine exerts lower toxicity and greater therapeutic effects against cerebral ischemic injury in vitro and in vivo than do H_2_S-releasing molecule alone or sulfide salt [60]. Some of H_2_S-releasing compounds have been tested in clinical trials [67–69]. Wallace and colleagues showed in a phase 2B clinical trial that naproxen chemically conjugated with a H_2_S-releasing moiety, ATB-346, inhibits COX-2 as well as naproxen with less gastrointestinal damage than naproxen (ClinicalTrials.gov Identifier: NCT03978208, NCT03291418) [67]. Sodium polythionate (SG1002) is being assessed the ability to elevate plasma H_2_S levels and to reduce markers of oxidative stress in heart failure patients in the phase II clinical trial (NCT01989208).

## Therapeutic effects of thiosulfate

In a series of experimental studies, we unexpectedly uncovered therapeutic effects of thiosulfate in models of critical illnesses. Thiosulfate has traditionally been considered as an inert end product of H_2_S oxidation. While sodium thiosulfate (STS) has been used as an antidote for cyanide poisoning, our discovery may expand its indication for other critical conditions.

**Fig. 3:**
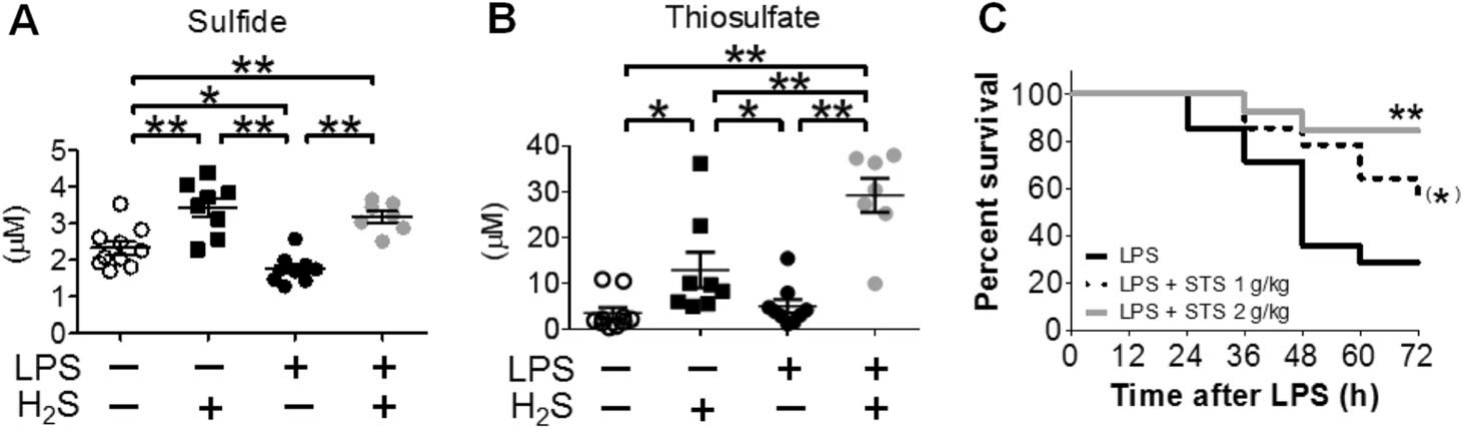
Plasma **a** sulfide and **b** thiosulfate concentration of mice after lipopolysaccharide (LPS) challenge followed by 6 h inhalation of air with or without breathing H_2_S (80 ppm) measured by monobromobimane-based high performance liquid chromatography (HPLC). *P < 0.05 and **P = 0.01, respectively. **c** Survival curve in mice challenged with LPS (LPS, *N* = 14), mice challenged with LPS and received 1 g/kg of STS (LPS + STS 1 g/kg, *N* = 14), and mice challenged with LPS and received 2 g/kg of STS (LPS + STS 2 g/kg, *N* = 13). **P = 0.0047 vs. LPS; *P = 0.0781 vs. LPS

We studied effects of inhaled H_2_S in a murine model of endotoxin-induced systemic inflammation and shock. Before our study, some studies showed pro-inflammatory effects of H_2_S whereas other studies reported anti-inflammatory effects of H_2_S. Our study revealed that endotoxin challenge decreased plasma sulfide concentration in mice. On the other hand, breathing H_2_S after endotoxin challenge restored sulfide levels and increased thiosulfate concentrations in plasma (Fig. 3a, b) that lead to attenuated systemic inflammation and improved survival of mice. The increased thiosulfate levels in endotoxin-challenged mice that breathed H_2_S appeared to be caused by endotoxin-induced upregulation of TST. Based on these observations, we hypothesized that thiosulfate may contribute to the beneficial effects of H_2_S inhalation. For the first time to our knowledge, we demonstrated that administration of STS dose-dependently improves survival rate of mice subjected to endotoxin challenge (Fig. 3c). These results put forth an innovative hypothesis that breathing H_2_S exerts anti-inflammatory effects and improves survival during murine endotoxin shock, in part by remodeling sulfide metabolism and increasing thiosulfate levels [70].

**Fig. 4:**
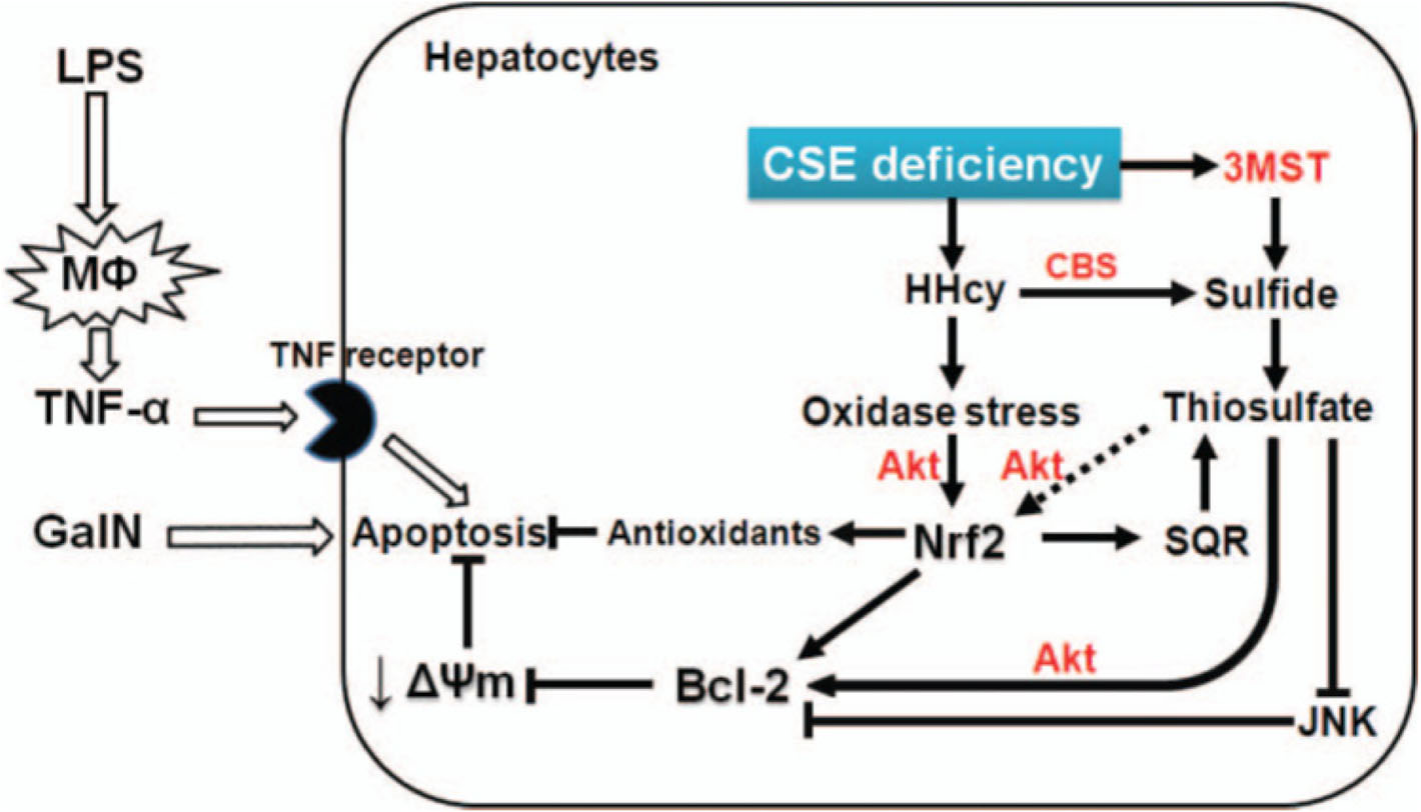
Hypothetical overview of hepatoprotective effects of CSE deficiency and thiosulfate on acute liver failure induced by GalN/LPS. M Φ macrophage, HHcy homocysteine, Akt protein kinase B, JNK c-Jun N-terminal kinase, Bcl-2 B cell lymphoma 2

To determine the role of endogenously produced H_2_S on inflammatory organ injury, we examined the outcomes of d-galactosamine (GalN)/lipopolysaccharide (LPS)-induced ALF in CSE-deficient mice on the C57BL6 background. A combination of GalN/LPS has been widely used to induce ALF in animal models. GalN sensitizes the liver toward other stimuli in part reflecting the role of uridine-containing compounds in hepatic biotransformation. Coadministration of LPS and GalN potentiates hepatic damage, leading to hepatocyte apoptosis. Given the protective effects of physiological levels of H_2_S against systemic inflammation, we hypothesized that CSE deficiency aggravates GalN/LPS-induced liver injury in mice. Unexpectedly, we observed that CSE deficiency attenuates liver injury and mortality in mice subjected to GalN/LPS-challenge, and prevents cell death in primary hepatocytes incubated with GalN/tumor necrosis factor (TNF)-α. Beneficial effects of CSE deficiency were associated with markedly elevated homocysteine and thiosulfate levels, upregulation of NF-E2 p45-related factor 2 (Nrf2) and antioxidant proteins, and markedly increased 3-MST and SQR expression in the liver. Upregulation of 3-MST seemed to compensate the decrease in sulfide production by CSE deficiency. Because upregulated 3-MST and SQR in CSE-deficient mice may accelerate H_2_S oxidation to thiosulfate, we again examined effects of STS in GalN/LPS-induced acute liver injury. We confirmed the robust cytoprotective effects of STS against acute liver failure (Fig. 4).

Another evidence that supports beneficial effects of thiosulfate came from our recent studies examining the mechanism of neuroprotective effects exerted by H2S donors. A number of studies suggest that H_2_S attenuates ischemia/reperfusion (I/R) injury in a variety of organs including the brain, whether it is endogenously produced or exogenously administered as H_2_S gas or donor compounds (typically Na_2_S or NaHS) [58–60, 71–73]. Nevertheless, mechanisms responsible for the cytoprotective effects of H_2_S were incompletely defined. In particular, since H_2_S has very short half-life in biological fluids including cell culture medium and blood, how H_2_S reaches its presumed targets in the cells, and in the target tissues in the body when given in vivo, has been poorly understood. In this study, we showed that H_2_S is mostly and quickly converted to thiosulfate in vitro and in vivo. While removal of thiosulfate from cell culture medium abolished the cytoprotective effects of Na_2_S against oxygen glucose deprivation, replacement of thiosulfate restored the protection. These results suggest that thiosulfate is not only required but sufficient for the cytoprotective effects of H_2_S. We observed that thiosulfate inhibits the mitochondrial apoptosis cascade and caspase-3 activity. The cytoprotective effects of thiosulfate were associated with increased persulfidation of cleaved caspase-3 at Cys^163^. The protective effect of Na_2_S or STS was facilitated by sodium sulfate cotransporter 2 (SLC13A4, NaS-2)-mediated transportation of thiosulfate across the cell membrane. Systemic administration of STS improved survival and neurological function of mice subjected to global cerebral I/R injury. Beneficial effects of STS, as well as Na_2_S, were associated with marked increase of thiosulfate, but not H_2_S, in plasma and brain tissues. These results suggest that thiosulfate is a circulating “carrier” molecule of cytoprotective effects of H_2_S.

Since STS is an inexpensive compound with low toxicity and proven safety track record of clinical use as an antidote for cyanide intoxication, STS is one of the most clinically relevant H_2_S- or reactive sulfur species-related compounds. STS has also been used to treat calciphylaxis, a potentially lethal complication of hemodialysis [74]. Effects of STS against ischemic heart diseases are currently examined in a clinical trial (NCT02899364). However, precise mechanisms responsible for the beneficial effects of STS in inflammation, ischemia-reperfusion, and calciphylaxis remain incompletely understood. Although our studies showed the possibility that thiosulfate itself may exert protective effects, it is also known that thiosulfate can be converted back to HS^−^ and persulfide/polysulfide directly or indirectly [75–77]. It is possible that several related sulfur molecules exert different and/or shared effects.

## Novel hydrogen sulfide scavengers to counter toxic effects of H_2_S

Hydrogen sulfide (H_2_S) is a highly toxic chemical hazard. Workers in industries including agriculture, petroleum, and sewage processing have been exposed to high concentration of H_2_S accidentally [1, 78]. As H_2_S can be easily and inexpensively made at home from materials found in local stores, it has been increasingly used for suicide [79, 80]. Among toxic gases, H_2_S is the second most common cause of death after CO [81]. Symptom of H_2_S poisoning varies depending on the gas concentration breathed. When H_2_S gas at 1000 ppm or higher concentration was inhaled, victims become unconscious and their respiratory center paralyzed instantly with only one or two breath. This is so-called knockdown and caused by instant paralysis of the central nervous system (CNS). One-time exposure to H_2_S can lead to long-term neurological deficits [82]. The US government considers H_2_S a high priority chemical threat, both industrially and as a potential weapon of mass destruction by terrorists. Mechanism of H_2_S poisoning is incompletely understood, and there is no antidote for H_2_S intoxication. Sodium nitrite, hydroxocobalamin, thiosulfate, hyperbaric oxygen, and hypothermia have been used after acute H_2_S poisoning with limited efficacy [81].

Toxic effects of H_2_S are caused not only by exogenous H_2_S but also by accumulation of endogenous H_2_S. Several observations indicate that H_2_S toxicity could be induced by disruption of endogenous H_2_S-production/catabolism balance. For example, ETHE1 deficiency is a cause of ethylmalonic encephalopathy. Cardinal features of ethylmalonic encephalopathy are associated with extreme elevation of circulating and tissue H_2_S levels [19]. H_2_S accumulates in hypoxic conditions due to the inhibition of SOU activity and decreased spontaneous oxidation [49, 83, 84]. CBS and CSE could translocate into mitochondria in hypoxic condition as described above. Therefore, hypoxia possibly causes H_2_S to accumulate to the toxic level in mitochondria. Qu et al. reported that accumulation of brain H_2_S during ischemia is a possible mediator of the brain damage after permanent focal cerebral ischemia in mice [65]. They demonstrated that the increase in brain H_2_S level is associated with cerebral ischemic injury and pre-ischemic inhibition of H_2_S-synthesis enzymes reduces cerebral infarct size. On the other hand, some reports have suggested the therapeutic effect of H_2_S-releasing compounds that are systemically administered early after reperfusion against cerebral ischemia/reperfusion [59, 60, 85, 86]. This apparent conflict about the role of H_2_S in ischemia/reperfusion might be explained by the dynamics of tissue H_2_S concentration during ischemia and after reperfusion as well as the narrow therapeutic window of H_2_S. Sulfide levels increase during ischemia and decrease after reperfusion in tissues, including brains [17, 65, 85, 87]. We and others reported that restoration of physiological sulfide levels mitigates I/R injury [58, 59, 85]. Although administration of low doses of sulfide donors at the time of or after reperfusion can activate several cytoprotective signaling cascades and attenuate reperfusion injury, slight overdose or delayed administration is often ineffective or harmful [58, 59, 85, 88]. Translation of sulfide-based therapy to clinical practice has been stymied due to the very low therapeutic index of sulfide [58, 60, 89] and the incomplete understanding of endogenous sulfide metabolism during ischemia and after reperfusion. Although keeping sulfide concentrations in the narrow therapeutic range appears to be critical, currently available pharmacological tools (e.g., inhibitors of H_2_S-producing enzymes) fail to achieve this goal. These observations prompted us to explore the role of sulfide catabolism in cellular respiration and survival.

**Fig. 5:**
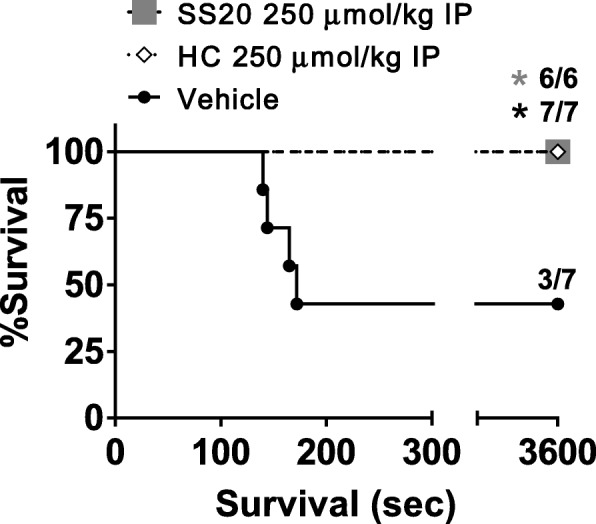
Survival rate curve of mice intoxicated with Na_2_S at 125 mg/kg IP. Vehicle (saline) or a H_2_S scavenger (SS20 or hydroxocobalamin, HC) was administered at 1 min after Na_2_S challenge. *P* < 0.05 vs. vehicle (saline)

To better understand the role of sulfide catabolism and potentially develop countermeasures against H_2_S poisoning, we recently launched a project to develop novel H_2_S-specific scavengers in collaboration with Xian laboratory [90]. To the best of our knowledge, specific H_2_S scavengers to control endogenous H_2_S levels have not been explored or reported. It should be noted that H_2_S scavengers are well-known in industrial settings as the removal of H_2_S or related sulfur-containing compounds in industrial processes has been extensively studied [91]. Materials like metallic oxide, alkanolamines, oxidizing chemicals, metal carboxylates/chelates, aldehydes, and triazines have been used as H_2_S scavengers. Unfortunately, these industrial sulfide scavengers cannot be applied into biological systems because of toxicity. Several compounds are known and used clinically as antidotes for H_2_S poisoning, but their specificity for H_2_S and applications for H_2_S-related pathologies have not been studied. For example, hydroxocobalamin (HC) has been investigated as an antidote for H_2_S poisoning, but it also scavenges cyanide, NO, CO, and ROS [92–94]. In our recently published study, we identified a series of sulfonyl azide compounds as promising H_2_S scavengers by exploiting the library of existing specific chemical H_2_S sensors and conducting extensive in vitro and in vivo screening. Sulfonyl azide compounds exhibit fast reaction time with H_2_S, high specificity against sulfide, low cellular toxicity, and capability to remove H_2_S in cellular systems. Systemic administration of SS20, one of these sulfonyl azide-based H_2_S scavengers, prevented death in mice subjected to acute H_2_S poisoning (Fig. 5) [90]. These results suggest that H_2_S scavengers may function as effective antidotes for H_2_S poisoning. Further studies are warranted to determine the effects of H_2_S scavengers in situations where endogenous H_2_S accumulation may be pathogenic.

## Conclusions

Intensive research in the last decade established that H_2_S is an important signaling molecule. Current knowledge indicates that dysregulated H_2_S levels are linked to a number of pathological processes including cancer, inflammation, diabetes, hypertension, and neurodegenerative diseases [95–98]. Consequently, chemical compounds that can be used to precisely regulate local H_2_S concentrations (both up and down) are important research tools as well as potential therapeutic agents. At the same time, it has become evident that currently available pharmacological tools are not sufficiently specific or versatile to elucidate the precise role of H_2_S in biology. Our discovery that thiosulfate may be an important carrier molecule of the biological effects of H_2_S may aid future research on the systemic effects of H_2_S. Recent development of specific H_2_S scavengers will enable more mechanistic studies by removing H_2_S from cellular milieu as well as propose a novel countermeasures against H_2_S poisoning. Because therapeutic window of H_2_S is narrow, to clarify how changes in balance of H_2_S production and catabolism play in illness must lead to further strategy of H_2_S-based therapies. This balance alteration seems to depend on the type of tissues and illness due to the diversity of expression levels related to H_2_S metabolism in tissues. For example, CNS is very sensitive to H_2_S poisoning due to the minimal level of H_2_S catabolizing capacity. Therefore, CNS seems to readily be affected by H_2_S toxicity in illness that increases H_2_S production [65, 99]. Further researches for H_2_S-production/catabolism balance in illness as well as development of novel pharmacological tools will undoubtedly advance our understanding of this fascinating gaseous molecule.

## Data Availability

Not applicable.

## Abbreviations

3-MST: 3-Mercaptopyruvate sulfurtransferase
ALF: Acute liver failure
AOAA: Aminooxyacetic acid
CARS: Cysteinyl-tRNA synthetase
CAT: Cysteine aminotransferase
CBS: Cystathionine β-synthase
CNS: Central nervous system
CO: Carbon monoxide
CoQ: Coenzyme Q
CSE: Cystathionine γ-lyase
ETC: Electron transport chain
ETHE1: Ethylmalonic encephalopathy 1
GalN: d-Galactosamine
GSH: Glutathione
GSSH: Glutathione persulfide
H_2_S: Hydrogen sulfide
HC: Hydroxocobalamin
I/R: Ischemia/reperfusion
LPS: Lipopolysaccharide
MetHb: Methemoglobin
NO: Nitric oxide
O_2_: Oxygen
PAG: dl-Propargyl glycine
PLP: Pyridoxal 5′-phosphate
SDO: Sulfide dioxygenase
SO: Sulfite oxidase
SOU: Sulfide oxidation unit
SQR: Sulfide quinone oxidoreductase
STS: Sodium thiosulfate
TNF-α: Tumor necrosis factor
TST: Rhodanese or thiosulfate sulfurtransferase
